# Supplementary material for: Sensory neurons regulate stimulus-dependent humoral immunity in mouse models of bacterial infection and asthma
Source: Nat Commun. 2024 Oct 16;15:8914. doi: 10.1038/s41467-024-53269-3 (PMC11484968; doi:10.1038/s41467-024-53269-3)
Supplement: Supplementary file 1 — Supplementary Information [file 41467_2024_53269_MOESM1_ESM.pdf]

Supplementary materials for

Sensory neurons regulate stimulus-dependent humoral immunity in mouse models of bacterial infection and asthma

Diane Aguilar*, Fengli Zhu*, Antoine Millet, Nicolas Millet, Patrizia Germano, Joseph Pisegna, Omid Akbari, Taylor A. Doherty, Marc Swidergall, Nicholas Jendzjowsky[1]

***Authors contributed equally**
1Correspondence to Nicholas Jendzjowsky (nicholas.jendzjowsky@lundquist.org)

This PDF file includes:
Supplementary figures
Materials list
References

Source data and materials are included in Source Data file.

Supplementary figure 1.

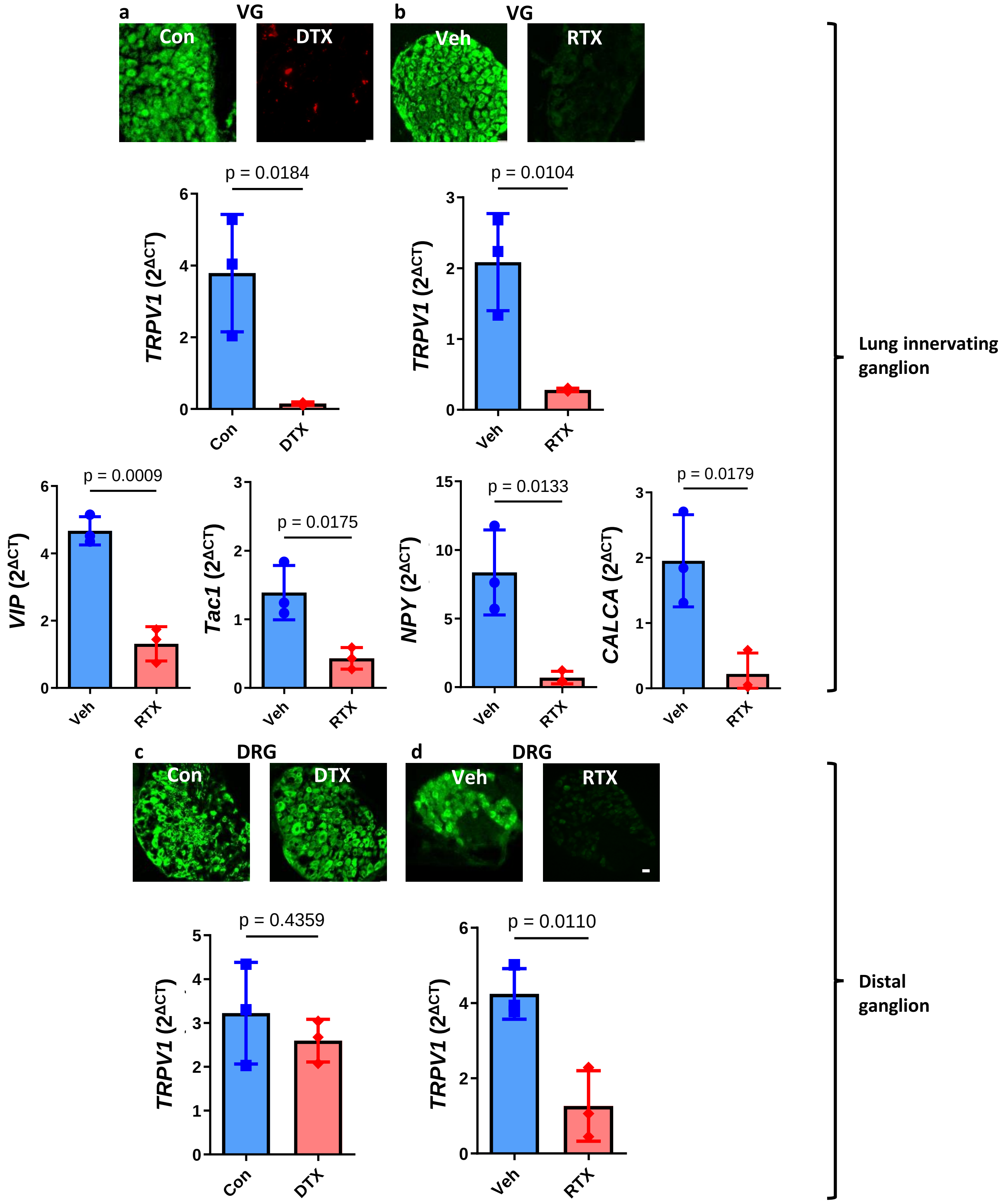

Supplementary figure 1. TRPV1 knockdown by direct vagal (VG) diphtheria toxin (DTX) injection or global sensory neuron ablation with subcutaneous resiniferatoxin (RTX) injection. a) qPCR demonstrates >85% downregulation of *TRPV1* gene from vagal (VG) ganglia in DTX 200ng in 200nl VG injected versus sham injected *TRPV1-DTR* mice. *TRPV1* is tagged with GFP in these mice, retrobeads were co-injected into *TRPV1-DTR* mice to demonstrate placement of injection as per Tränkner et al. In sham mice, the vagal ganglia were identified, and vehicle was injected in the vicinity of the VG. The prominent neuropeptides- vasoactive intestinal peptide (*VIP*), substance P (denoted by *Tac1* gene), calcitonin gene related peptide (denoted by *CALCA* gene) and neuropeptide Y (*NPY*) were all down-regulated in the nodose/jugular ganglion with RTX treatment). **b)** *TRPV1* gene is >85% downregulated in RTX compared to vehicle-injected mice in VG. **c)** *TRPV1* is maintained in dorsal root ganglia (DRG) of VG DTX injected mice compared to sham injected mice. **d)** *TRPV1* is downregulated in DRG with global RTX injection. Two-sided t-test of ΔCT values. Housekeeping gene = hypoxyribosyltransferace. N=3 mice per sample (6 ganglia per sample), N=3 samples per group and condition.

Supplementary figure 2.

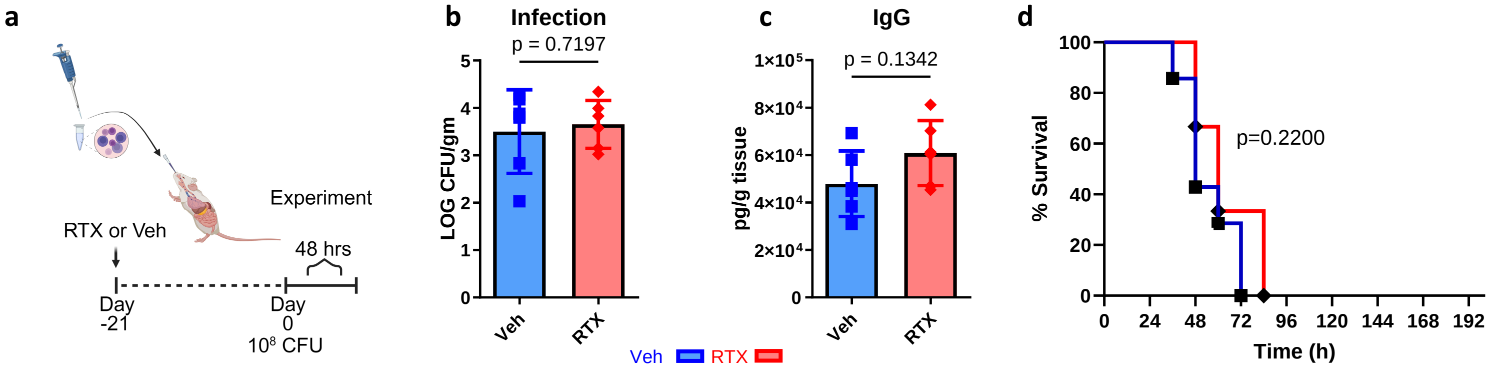

Supplementary figure 2. Sensory neuron ablation does not alter bacterial clearance following acute infection with *S. pneumoniae*. a) A single dose of 10^8 CFU *S. pneumoniae (Serotype 19F, ATCC49619)* was delivered after treatment with vehicle or RTX as per our sensory neuron ablation model and then lungs were harvested to assess bacterial burden and immunoglobulin concentration 48h after infection. Image created in BioRender. Aguilar, D. (2022) BioRender.com/h64o602. **b**) Log CFU per gram lung mass from acute infected sensory neuron intact (Veh) or sensory neuron ablated mice(RTX) show a similar bacterial burden. **c**) IgG concentrations from lung homogenates were similar between Veh and RTX 48h after acute infection. Veh: n=6, RTX: n=6. Two-sided T-test. **d**) A single dose of 10^6 CFU *S. pneumoniae (Serotype 3, ATCC 6303)* in 50µl PBS was delivered after treatment with vehicle or RTX as per our sensory neuron ablation model and then survival was assessed. Veh: n=7, RTX: n=6. Data were compared with Log-rank (Mantel-Cox) test.

Supplementary figure 3.

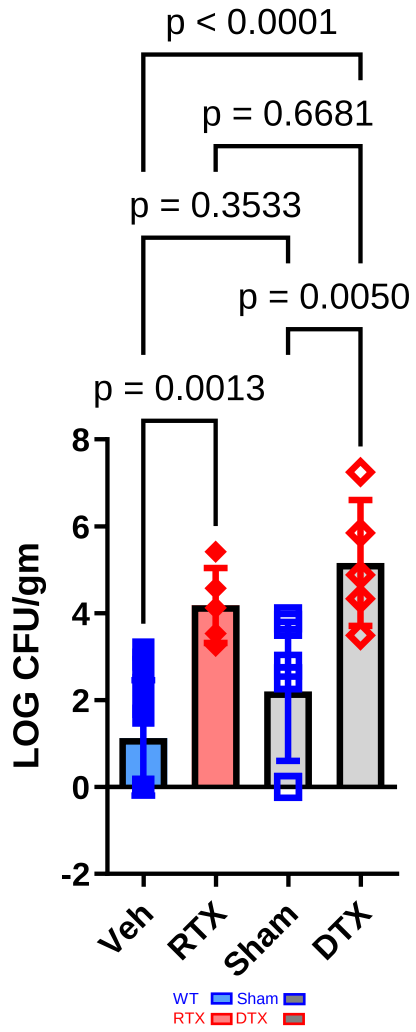

Supplementary figure 3. The effect of bacterial clearance by sensory neurons is specific to vagal ganglia. We compared the method of sensory neuron ablation on disease outcome. The bacterial burden in *TRPV1-DTR* vagal ablated or RTX mice was similarly increased 48h after the final infection with 10^8 CFU of *S. pneumoniae (Serotype 19F, ATCC49619)* in comparison to Sham injection or Veh, respectively. Model of *S. pneumoniae* pre-exposure and infection as per Figure 1. Veh: n=13, RTX: n=5. Sham: n=7, DTX: n=5. One-way ANOVA with Tukey's post hoc test.

Supplementary figure 4.

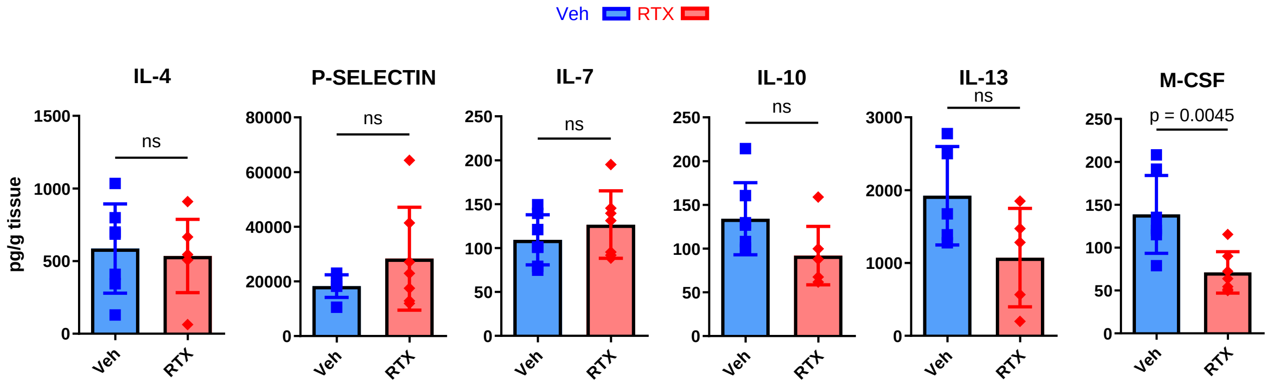

Supplementary figure 4. Cytokines unaffected by sensory neuron ablation in response to pre-exposure and infection with *S. pneumoniae*. Quantification of IL-4, P-Selectin, IL-7, IL-10, IL-13, M-CSF in sensory neuron intact (Veh, n=7) and sensory neuron ablated (RTX, n=6) mice 16h following the final 10^8 CFU dose of *S. pneumoniae*. Two-sided t-test. Data were pooled from 2 independent experiments.

Supplementary figure 5.

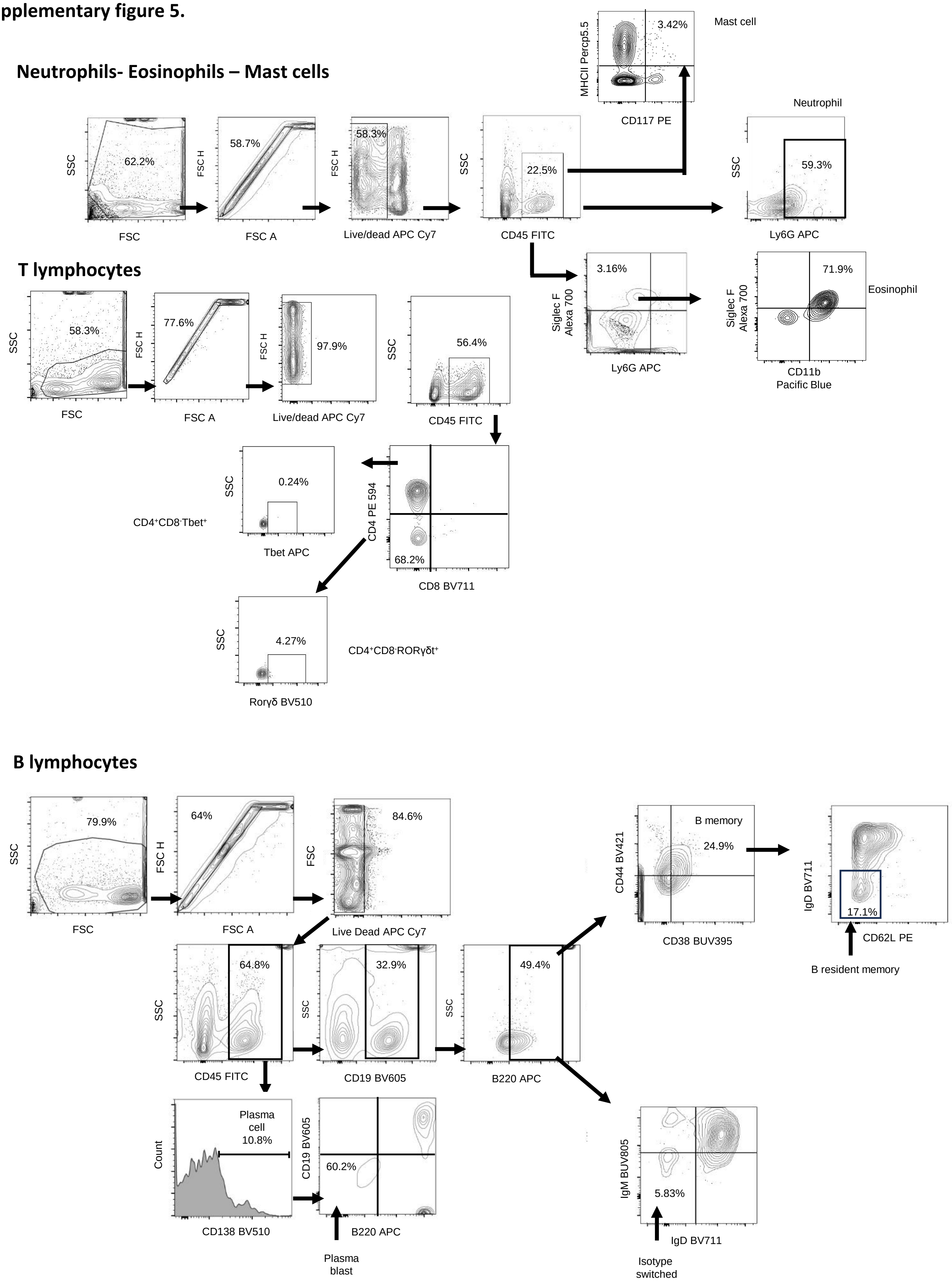

Supplementary Figure 5. Gating strategy for Neutrophils, Eosinophils, Mast cells, T lymphocytes and B lymphocytes. Gating used for *in vivo* flow cytometry data from lungs, spleen and bone marrow collected on BD Symphony A5.

Supplementary figure 6.

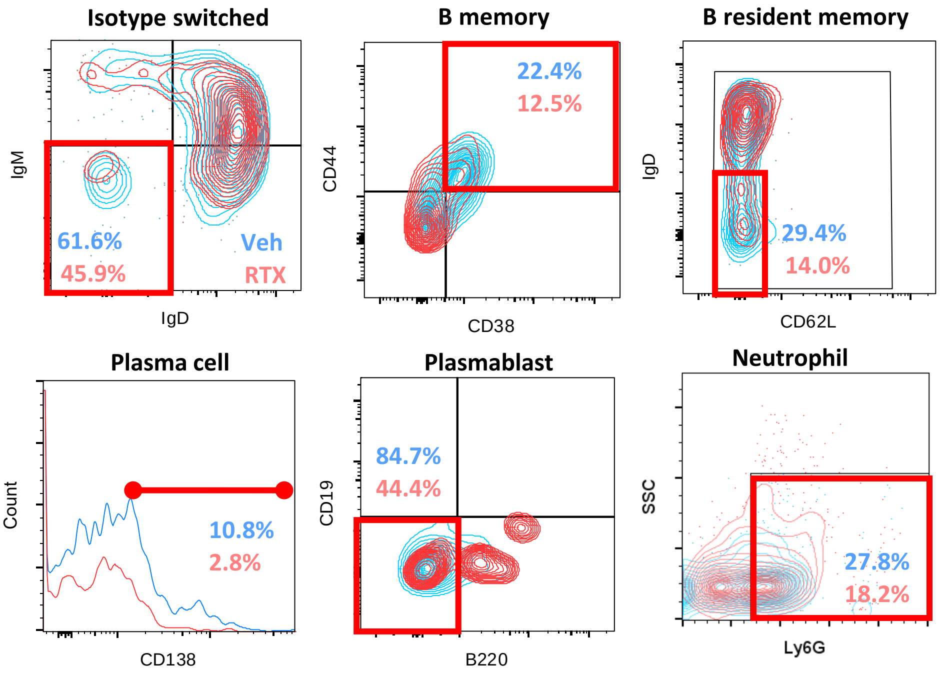

Supplementary Figure 6. Comparisons B cell populations between sensory neuron intact and depleted mice at 48h after final infection which was preceded with pre-exposure. Gating used for *in vivo* flow cytometry data for B cell populations. Vehicle treated sensory neuron intact mice (Veh) are in blue and sensory neuron depleted mice (RTX) are in red. The representative gate for Isotype Switched, B memory, B resident memory, Plasma cell, Plasmablast and Neutrophils are shown in red gates. Populations were gated as per Supplementary Figure 5. Collected on BD Symphony A5.

Supplementary figure 7.

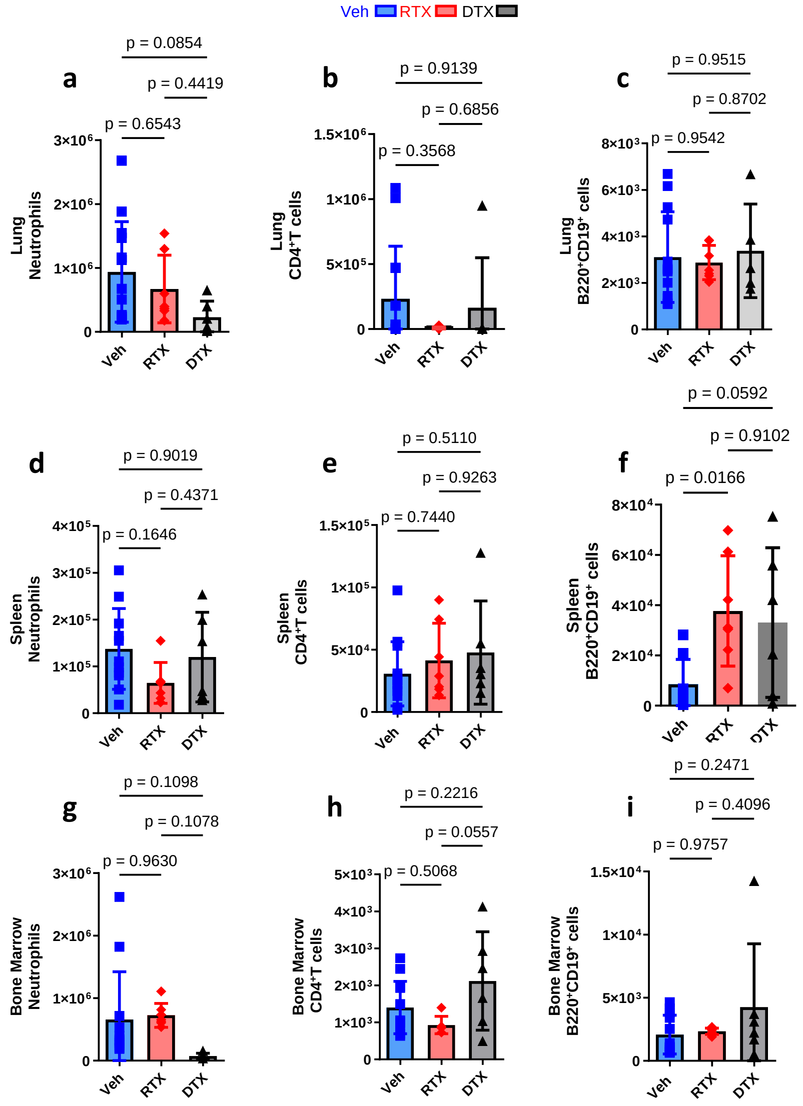

Supplementary Figure 7. Sensory neuron ablation does not effect bone marrow or spleen cells. a-c) Lung, **d-f)** Spleen and **g-i)** Bone Marrow populations of **a, d, f)** Neutrophils (CD45$^+$Ly6G$^+$), **b, e, h)** T helper lymphocytes (CD45$^+$CD4$^+$CD8$^-$) or **c, f, i)** B lymphocytes (CD45$^+$B220$^+$CD19$^+$ all gated on live singlets) were maintained in both conditions 16h following pre-exposure and infection with *Streptococcus Pneumoniae 19F*. DTX and RTX models showed similarities with cell populations. Total cell populations were determined with counting beads. Veh: n=13, RTX: n=7, DTX: n=6. Data from 3 independent experiments. One-way ANOVA with Tukey's post hoc test.

Supplementary figure 8.

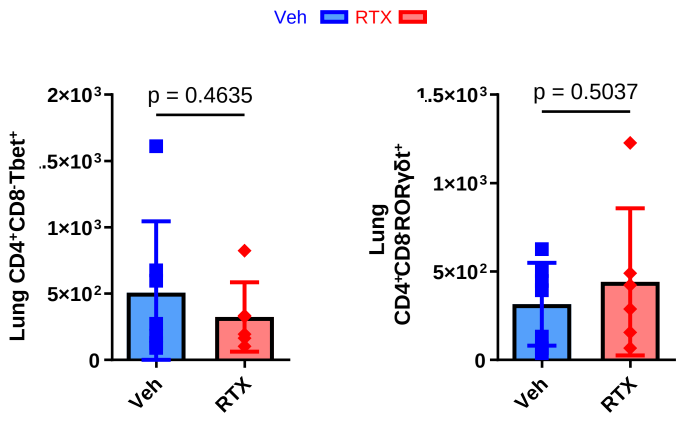

Supplementary figure 8. Sensory neuron ablation does not reduce T lymphocytes 48h after infection with *S. pneumoniae*. T helper cells were similar in sensory neuron intact and ablated mice. Vehicle: n=6, RTX: n=6. Cells were gated on live CD45+ cells. Counting beads determined cell populations. Data from 2 independent experiments. Two-sided t-test.

Supplementary figure 9.

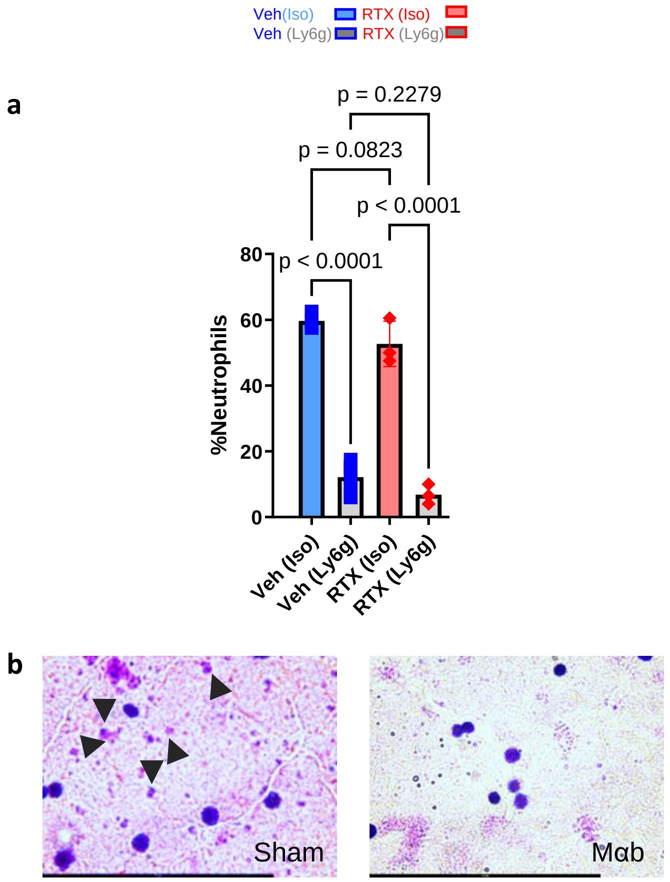

Supplementary figure 9. Neutrophil depletion. a) Circulating neutrophils were suppressed with anti-1A8 antibody. Veh (Iso): n=7, RTX (Iso):n=3, Veh (Ly6G): n=8, RTX (Ly6G): n=3. Wight-Giemsa stain on blood smear. One-way ANNOVA with Tukey's post-hoc test. Data from 2 independent experiments. **b**) Representative blood smears from Isotype treated and neutrophil depletion treated mice. Arrowheads point to neutrophils. Scalebar=50um.

Supplementary figure 10.

a

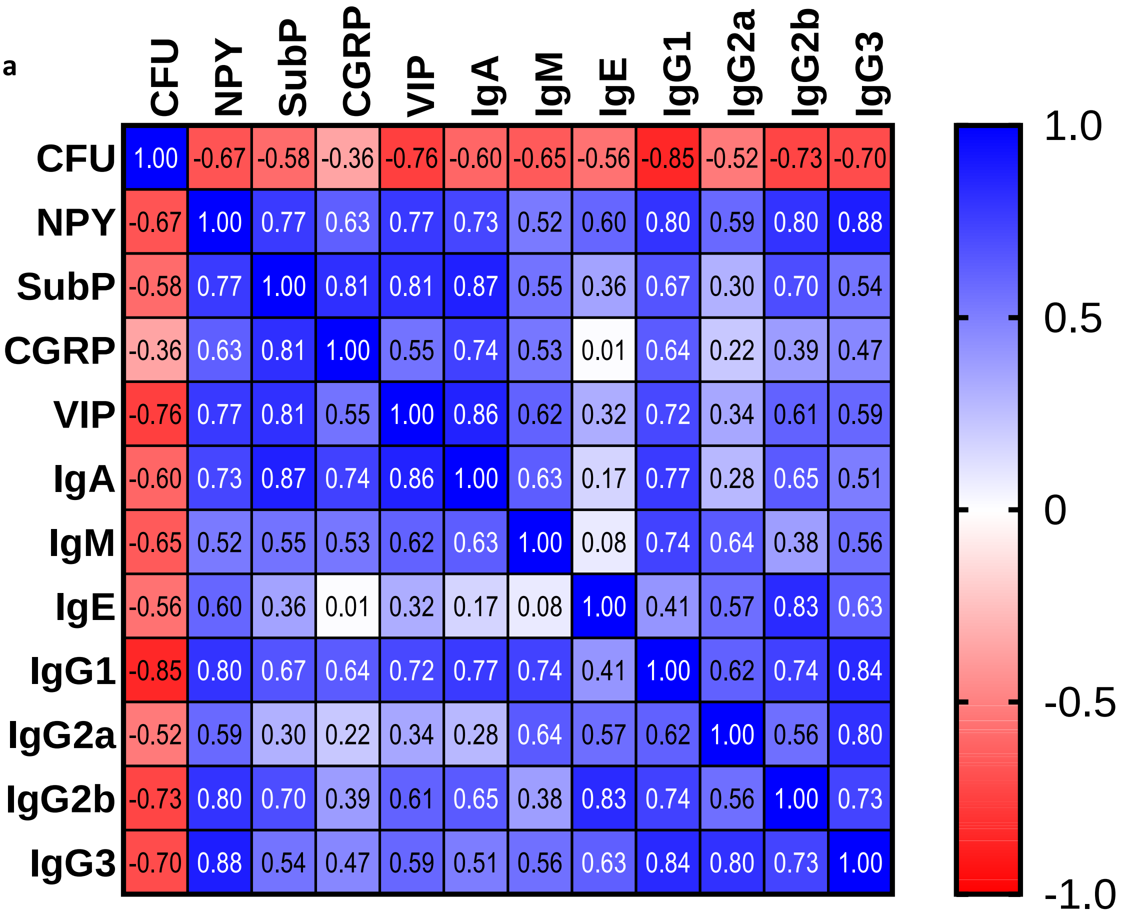

b

	CFU	NPY	SubP	CGRP	VIP	IgA	IgM	IgE	IgG1	IgG2a	IgG2b	IgG3
CFU		0.01653	0.04710	0.24975	0.00438	0.03758	0.02219	0.06014	0.00045	0.08170	0.00667	0.01060
NPY	0.01653		0.00326	0.02953	0.00320	0.00762	0.08230	0.04060	0.00180	0.04487	0.00194	0.00013
SubP	0.04710	0.00326		0.00130	0.00142	0.00022	0.06640	0.25111	0.01760	0.33736	0.01195	0.06704
CGRP	0.24975	0.02953	0.00130		0.06616	0.00577	0.07582	0.97280	0.02450	0.50163	0.21350	0.12286
VIP	0.00438	0.00320	0.00142	0.06616		0.00030	0.03329	0.31057	0.00884	0.27689	0.03333	0.04195
IgA	0.03758	0.00762	0.00022	0.00577	0.00030		0.02699	0.60501	0.00341	0.38203	0.02330	0.09246
IgM	0.02219	0.08230	0.06640	0.07582	0.03329	0.02699		0.80710	0.00605	0.02400	0.22491	0.05849
IgE	0.06014	0.04060	0.25111	0.97280	0.31057	0.60501	0.80710		0.18249	0.05487	0.00080	0.02906
IgG1	0.00045	0.00180	0.01760	0.02450	0.00884	0.00341	0.00605	0.18249		0.03256	0.00545	0.00066
IgG2a	0.08170	0.04487	0.33736	0.50163	0.27689	0.38203	0.02400	0.05487	0.03256		0.05679	0.00159
IgG2b	0.00667	0.00194	0.01195	0.21350	0.03333	0.02330	0.22491	0.00080	0.00545	0.05679		0.00709
IgG3	0.01060	0.00013	0.06704	0.12286	0.04195	0.09246	0.05849	0.02906	0.00066	0.00159	0.00709	

Supplementary figure 10. Neuropeptides and immunoglobulins correlate with bacterial burden. Lung bacterial burden (Figure 1b- 48h timepoint), immunoglobulins (Figure 2a) and neuropeptides (Figure 3b, c, d, e) were compared with Pearson correlation. CFU was inversely correlated with neuropeptides and immunoglobulins with VIP and IgG1 showing the strongest relationships to Lung bacterial burden (CFU). Neuropeptides and immunoglobulins were positively correlated with one another. **a**) Pearson r. **b**) P-values.

Supplementary figure 11.

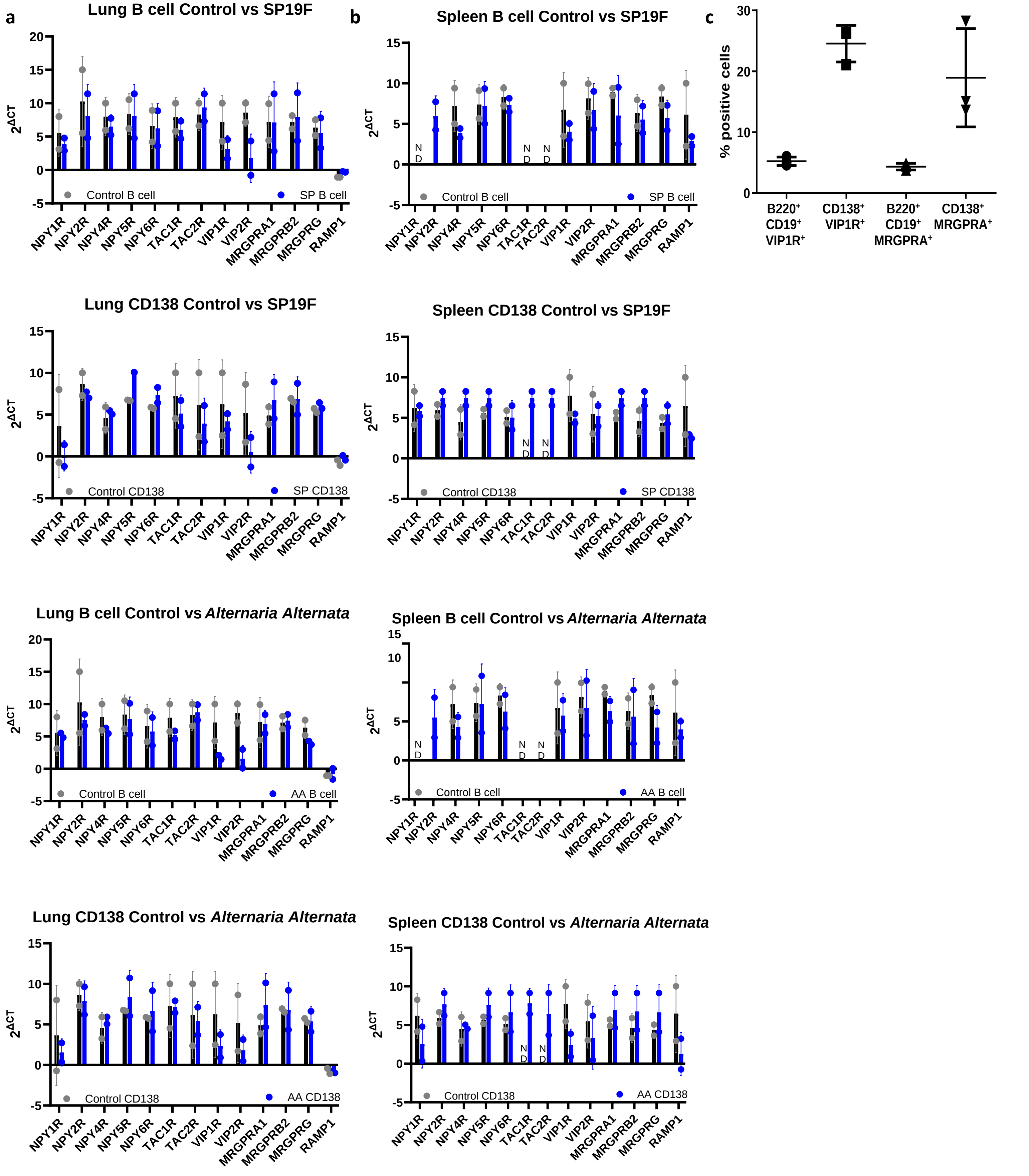

Supplementary figure 11. Prominent neuropeptide receptors in isolated B cells. CD138+ and B cells were isolated from the lungs (**a**) and spleen (**b**) of naïve, *S. pneumoniae* and *A. Alternata* mice (n=5 mice were pooled per sample, 2 samples per group). Cells were isolated with the CD138 positive or B cell negative EasySep selection kit. qPCR for prominent neuropeptide receptors was run, and data were expressed as ΔCT from the hypoxyribosyltransferase housekeeping gene. No differences between groups within the respective subset were demonstrated for any receptor. Although we note that *VIPR1, VIPR2, RAMP1* are highly expressed in all samples. **c**) Protein surface expression from splenic naïve isolated B cells were gated on Live B220$^+$CD19$^+$ or CD138$^+$ cells. VIPR1 and MRGPRG were highly expressed on plasma cells. N=3.

Supplementary figure 12.

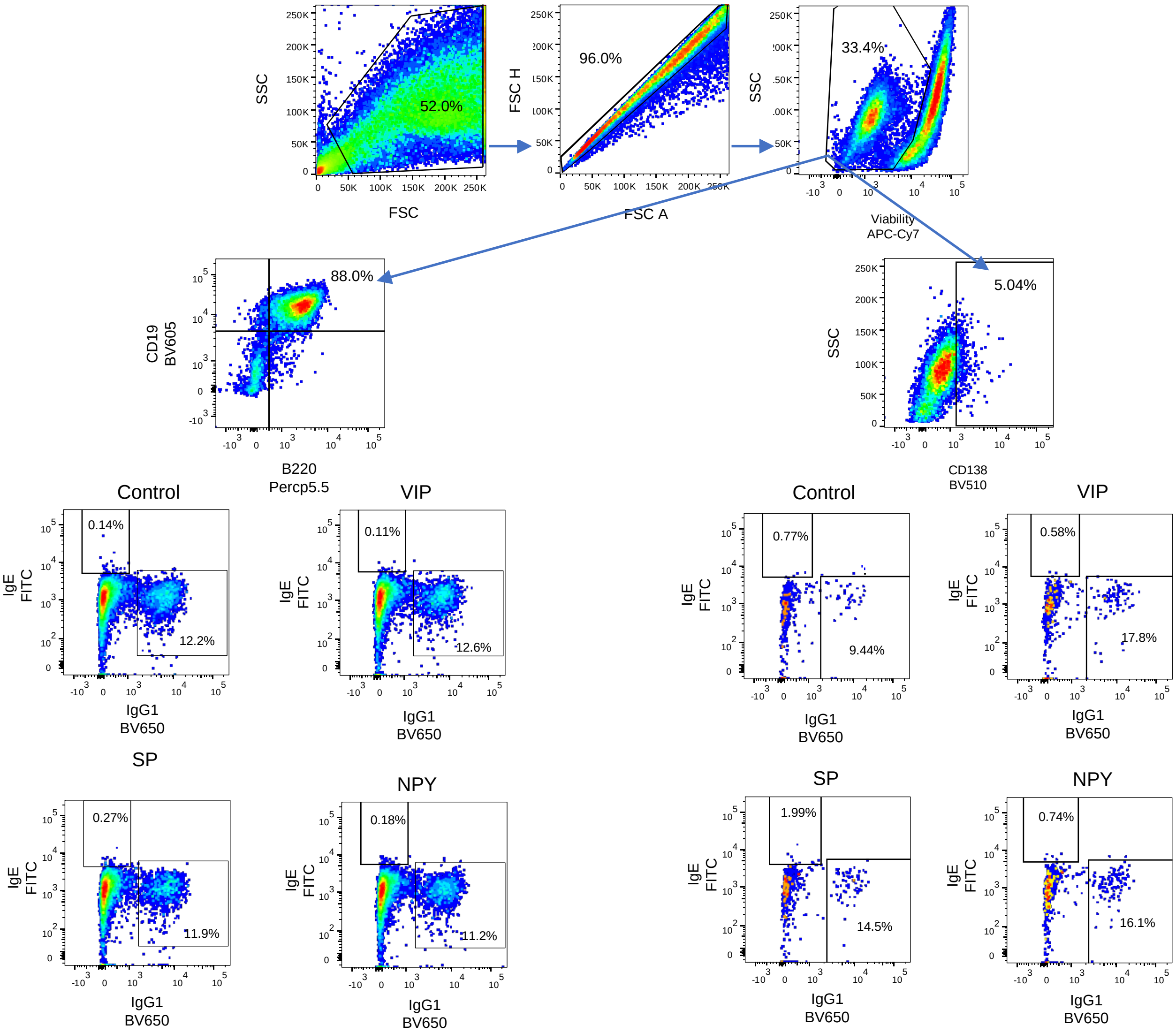

Supplementary Figure 12. Comparisons of neuropeptide stimulated cultured B cells. Gating used for *in vitro* flow cytometry data for B cell populations. Collected on BD Symphony A5.

Supplementary figure 13.

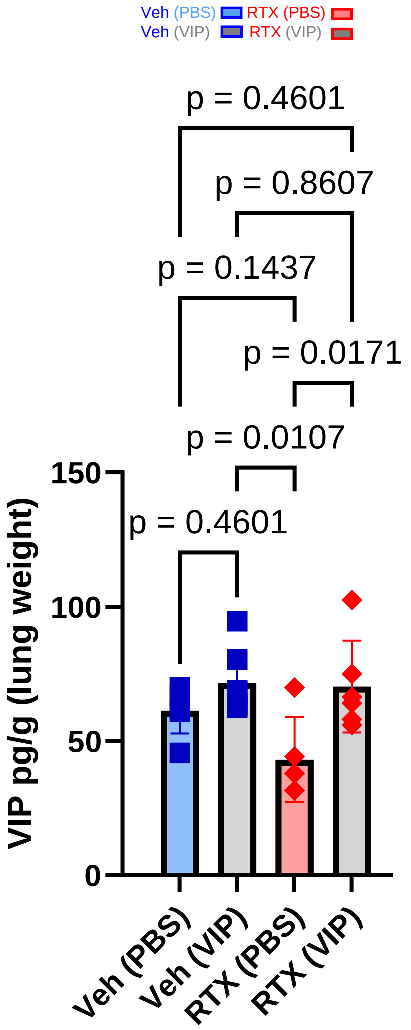

Supplementary figure 13. Vasoactive intestinal peptide supplementation increases vasoactive intestinal peptide in RTX mice. Mice were treated as per Figure 4a. VIP was measured from lung homogenates and expressed per lung weight. VIP supplementation significantly increased VIP levels in lungs of RTX mice 16hrs after final infectious dose of bacteria. Veh (PBS): n=6, Veh (VIP): n=7, RTX(PBS): n=5, RTX(VIP): n=6. One-way ANOVA with Holm Sidak post hoc test. Data from 3 independent experiments.

Supplementary figure 14.

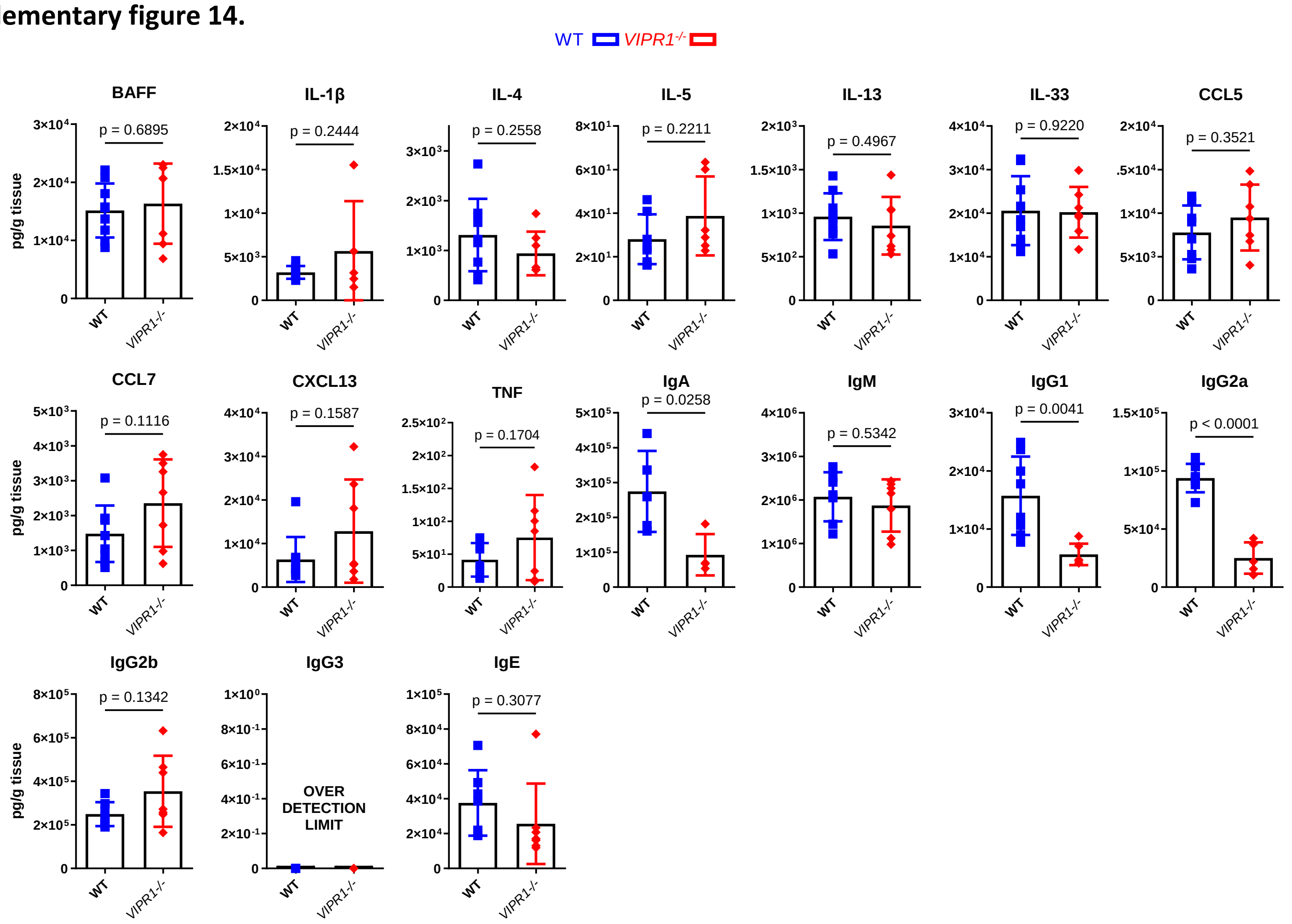

Supplementary figure 14. VIP 1 receptor knockout mice have reduced immunoglobulins, but not cytokines compared to WT mice following pre-exposure and infection to *S. pneumoniae.* Quantification of BAFF, IL-1β, IL-4, IL-5, IL-13, IL-33, CCL5, CCL7, CXCL13, TNF, IgA, IgM, IgG1, IgG2a, IgG2b, IgG3, IgE in WT and VIP 1 receptor knockout mice (*VIPR1-/-*) following pre-exposure and infection to *S. pneumoniae* WT: n=6-8, *VIPR1-/-*: n=5-7. Two-sided t-test. Data from 2 independent experiments.

Supplementary figure 15.

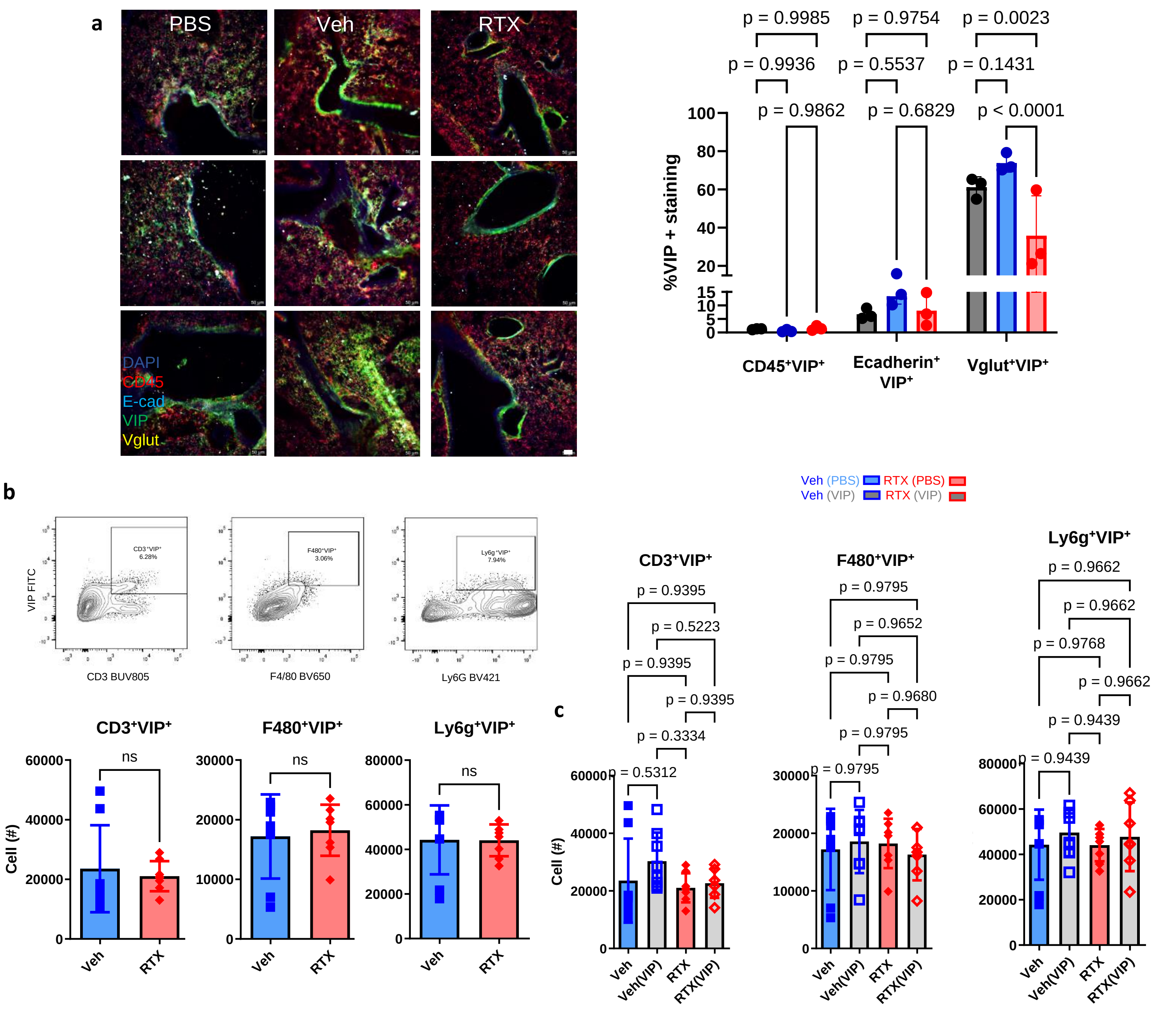

Supplementary figure 15. VIP from non-neuronal sources is similar between vehicle and RTX infected mice. a) *Vglutcre-tdtomato* mice underwent our pre-exposure/infection model as per Figure 1a. Lungs were harvested and stained for CD45 (leukocytes), E-cadherin (epithelium) and VIP. The VIP fluorescence signal was normalized to the CD45, Ecadherin or *Vglut* fluorescence signal. RTX only reduced VIP in *Vglut-expressing* cells. Two-way ANOVA with Holm-Sidak post-hoc test. N=3 per group. Scalebar= 50µm. **b**) Lungs from Veh or RTX mice were processed for flow cytometry. Cells were gated from CD45 live, singlets. RTX and Vehicle exposed to our pre-exposure and infection model as per Figure 1 had similar VIP expression in T-cells (CD3), Macrophages (F480) and neutrophils (Ly6g). N=8 per group. Two-sided t-test. **c**) Veh and RTX mice underwent VIP supplementation as per Figure 4a. Cells were stained as per panel b. No differences were demonstrated between groups for VIP from leukocytes. N=7-8 per group, One-way ANOVA with Holm-Sidak post-hoc test. Two independent experiments.

Supplementary figure 16.

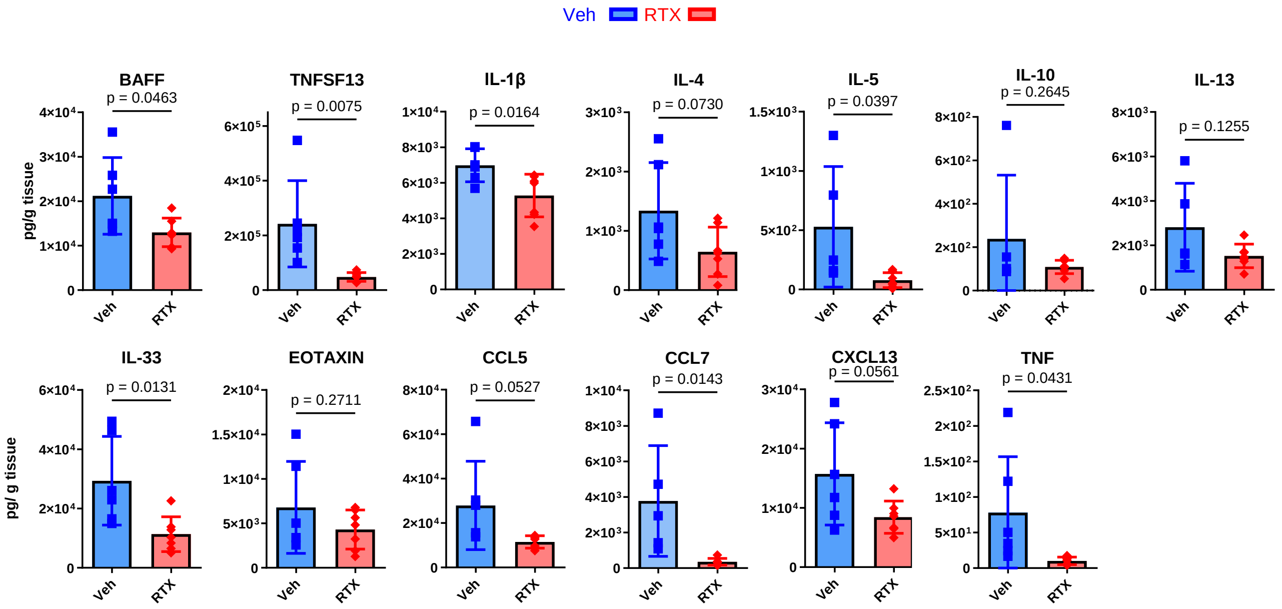

Supplementary figure 16. Sensory neuron ablation suppresses inflammatory, b-cell survival and recruiting cytokines and chemokines and Th2 cytokines following *A. alternata treatment*. Quantification of BAFF, TNFSF13, IL-1β, IL-4, IL-5, IL-10, IL-13, IL-33, Eotaxin, CCL5, CCL7, CXCL5 and, TNF in sensory neuron intact and sensory neuron ablated (RTX) after the final dose of *A. alternata*. Veh: n=5-7, RTX: n=6-7. Two-sided t-test. Data from 3 independent experiments.

Supplementary figure 17.

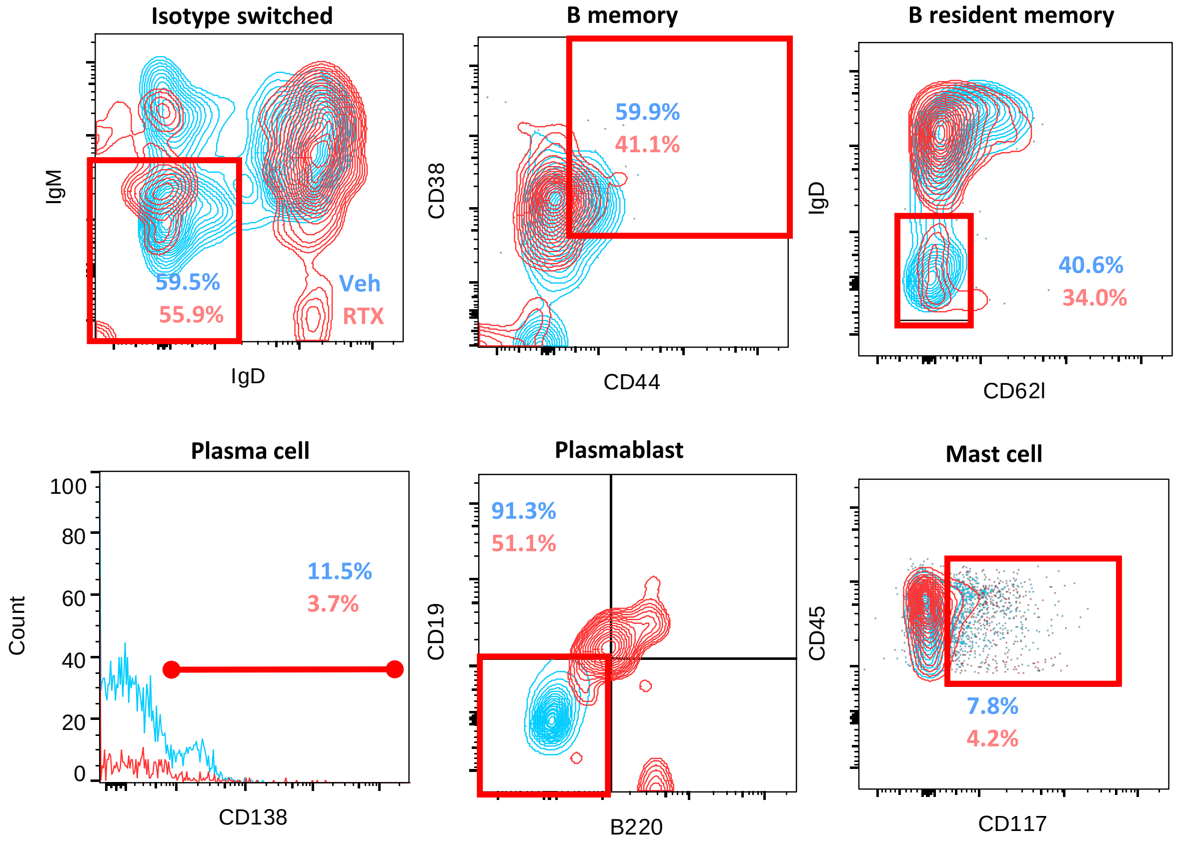

Supplementary Figure 17. Comparisons between sensory neuron intact and depleted mice with *Alternaria Alternata* asthma 16h after the last dose of *Alternaria Alternata*. Gating used for in vivo flow cytometry data for B cell populations. Vehicle treated sensory neuron intact mice are in blue and RTX sensory neuron depleted mice are in red. The representative gate for Isotype Switched, B memory, B resident memory, Plasma cell, Plasmablast and Mast cells are shown and gated as per Supplementary Figure 5. Collected on BD Symphony A5.

Supplementary figure 18.

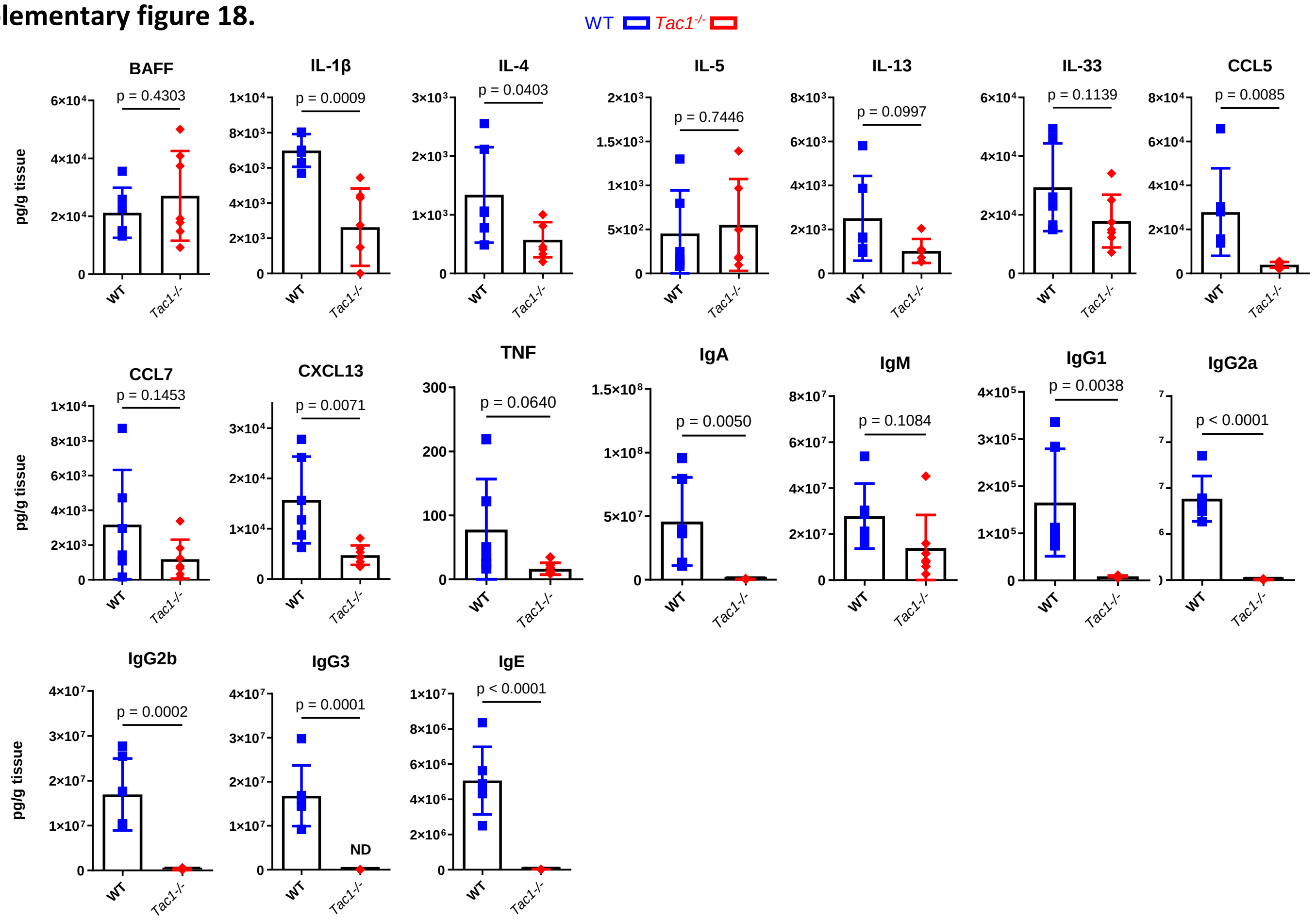

Supplementary figure 18. *Tac1* knockout mice have reduced inflammatory, b-cell survival, recruiting cytokines, chemokines, Th2 cytokines and immunoglobulins following *A. alternata treatment*. Quantification of BAFF, IL-1β, IL-4, IL-5, IL-13, IL-33, CCL5, CCL7, CXCL13, TNF, IgA, IgM, IgG1, IgG2a, IgG2b, IgG3, IgE (repeated from figure 6) in Wild type and *Tac1-/-* (substance P gene) mice after the final dose of *A. alternata*. WT: n=7-8, *Tac1-/-*: n=7-8. Two-sided t-test. Data from 2 independent experiments.

Supplementary figure 19.

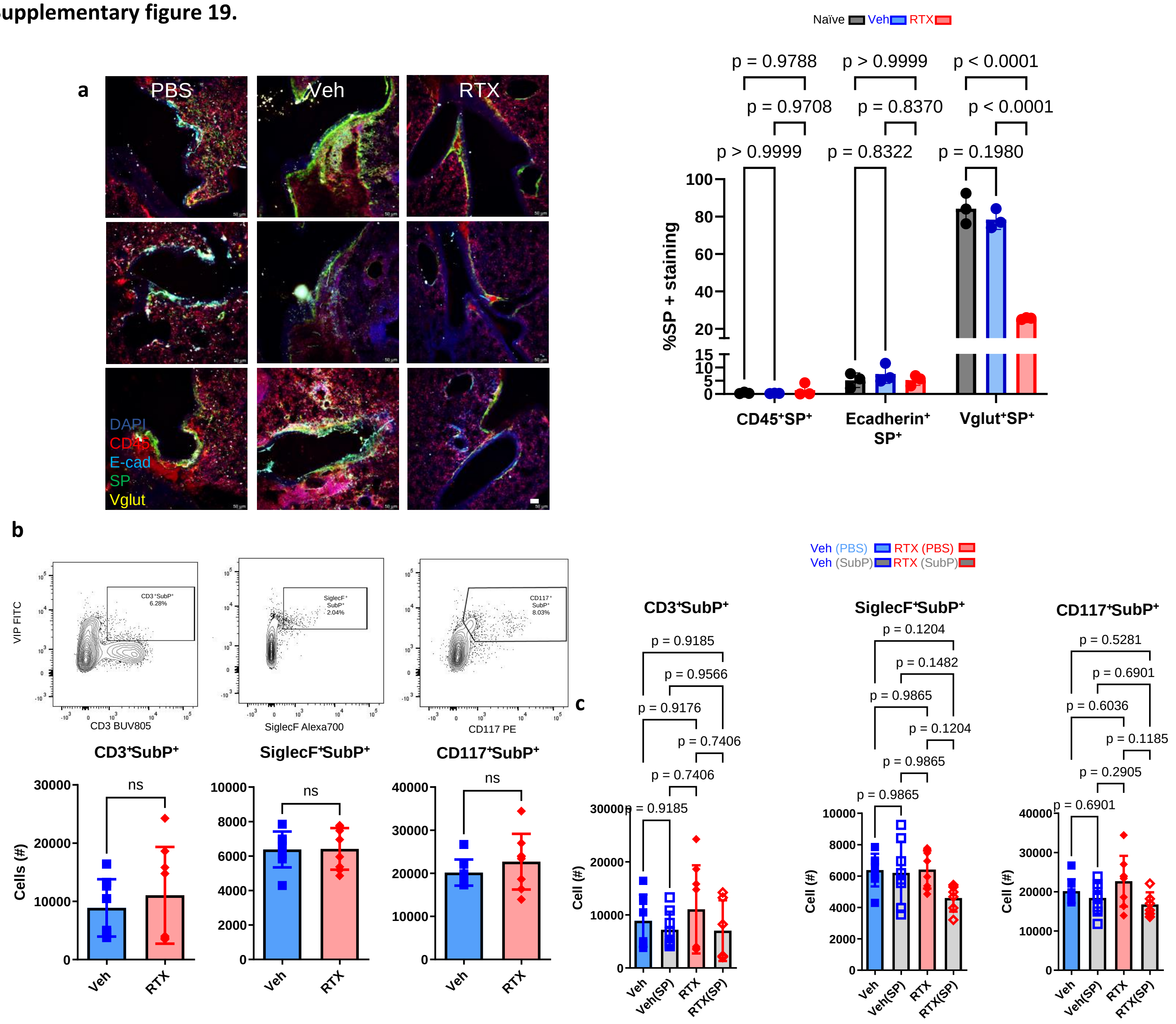

Supplementary figure 19. Substance P from non-neuronal sources is similar between vehicle and RTX *A. alternata* mice. a) Vglutcre-tdtomato mice underwent A Alternata as per Figure 5. Lungs were harvested and stained for CD45 (leukocytes), Ecadherin (epithelium) and Substance P (SubP). The SubP fluorescence signal was normalized to the CD45, Ecadherin or Vglut fluorescence signal. RTX only reduced VIP in Vglut expressing cells. Two-way ANOVA with Holm-Sidak post-hoc test. N=3 per group. Scalebar= 50µm. **b**) Lungs from Veh or RTX mice were processed for flow cytometry. Cells were gated from CD45 live, singlets. RTX and Vehicle exposed to our pre-exposure and infection model as per Figure 5 had similar SubP expression in T-cells (CD3), eosinophils (Siglec F) and mast cells (CD117). N=8 per group. Two-sided t-test. **c**) Mice underwent SubP supplementation as per Figure 6a. Cells were stained as per panel b. No differences were demonstrated between groups for SubP from leukocytes. N=6-8 per group, One-way ANOVA with Holm-Sidak post-hoc test. Two independent experiments.

Supplementary figure 20.

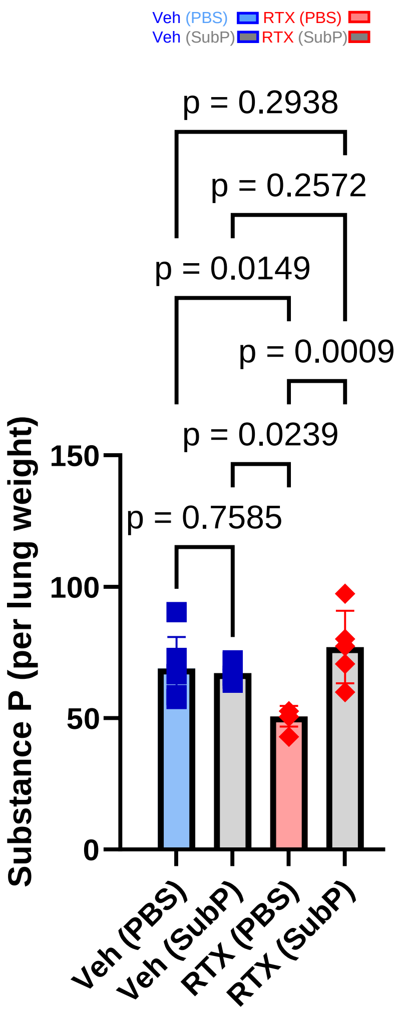

Supplementary figure 20. Substance P supplementation increases Substance P in RTX mice. Mice were treated as per Figure 6a. Substance P was measured from lung homogenates and expressed per lung weight. Substance P supplementation significantly increased Substance P levels in lungs of RTX mice 16hrs after final *A. alternata* dose. Veh (PBS): n=6, Veh (SubP): n=6, RTX(PBS): n=6, RTX(SubP): n=5. One-way ANOVA with Holm sidak post hoc test. Data from 3 independent experiments.

Table 1. Experimental models: organisms and strains

Experimental Models: Organisms and Strains			
**Strain**	**Supplier**	**Reference**	**Cat #**
C57BL/6	Jackson Laboratory		000664
B6.129S2-Ighmtm1Cgn/J	Jackson Laboratory		002288
B6J.129S6(FVB)-Slc17a6tm2(cre)Lowl/MwarJ	Jackson Laboratory		028863
B6.Cg-Gt(ROSA)26Sortm14(CAG-tdTomato)Hze/J	Jackson Laboratory		007914
TRPV1-DTR	Mark Hoon Creator; Donated by Isaac Chiu	PMID: 23536068; PMID: 29505031	
Vipr1tm1Msod/J	James Waschek (UCLA)	PMID: 21697765	MGI:J:177616
B6.Cg-Tac1tm1Bbm/J	Jackson Laboratory		#004103

Table 2: Antibodies

	Antibodies					
AB	Fluorophore	Clone	Vendor	Cat#	Lot#	Dilution
CD11c	BV 421	N418	BIOLEGEND	117329	B366519	1:100
Ly-6G	APC	1A8	BIOLEGEND	127614	B366717	1:100
Ly-6G	BV421	RB6-8C5	BIOLEGEND	108433	B320343	1:100
CD11B	PACIFIC BLUE	M1/70	BIOLEGEND	101224	B375478	1:100
MHCII	percp 5.5	M5/114.15.2	BIOLEGEND	107626	B354662	1:100
CD117 ckit	PE	2B8	BIOLEGEND	105808	B343465	1:100
Siglec-F	ALEXA 700	1RNM44N	INVITROGEN	561702-82	2410787	1:100
F4 80	BV650	BM8	BIOLEGEND	123149	B326894	1:100
CD3	BUV805	17A2	BDBiosciences	569192	3047446	1:100
CD4	PEC594	GK1.5	BIOLEGEND	100455	B333712	1:100
CD8	BV711	SK1	BIOLEGEND	344734	B370263	1:100
T-BET	APC	4B10	BIOLEGEND	644814	B353939	1:100
RORYT	BV510	Q31-378	BDBiosciences	567177	3027175	1:100
B220	PercP 5.5	RA3-6B2	BIOLEGEND	103 236	B358603	1:100
B220	APC	RA3-6B2	BIOLEGEND	103212	B379792	1:100
CD19	BV605	6D5	BIOLEGEND	115539	B386168	1:100
L-SELECTIN CD62L	PE	MEL-14	BIOLEGEND	104408	B374825	1:100
CD44	BV421	IM7	BIOLEGEND	103040	B393548	1:100
CD38	BUV395	90	INVITROGEN	363-0381-82	2774971	1:100
SYNDECAN -1 CD138	BV510	281-2	BIOLEGEND	142521	B374400	1:100
CD23	PE-Cy7	B3B4	BIOLEGEND	101614	B407653	1:100
IgD	BV711	11-26C.2a	BIOLEGEND	564275	3069422	1:100
IgG1	BV650	RMG1-1	BIOLEGEND	406629	B364333	1:100
IgM	BUV805	IL/41	BDBiosciences	749307	3219714	1:100
IgE	ALEXA 488	RME-1	BIOLEGEND	406909	B368990	1:100
Ki67	BUV737	SolA15	INVITROGEN	367-5698-82	2653121	1:100
CD45	ALEXA 488	30-F11	BIOLEGEND	103122	B369434	1:100
Viability	APC CY7		Invitrogen	65-0865-18	2836722	1:1000
CD16 32	TruStainFcx	93	BIOLEGEND	101320	B419152	1:100
VIP1R	FITC	AB_2341081	Alomone	AVR-001-F-50UL	AVR001FAN0150	1:100
TRPV1		AB_2313819	Alomone	ACC-030	ACC030AG0440	1:200
MRGPRX	PE-Cy7	K125H4	BIOLEGEND	359007	B393291	1:100
Tac1	FITC	polyclonal	Proteintech	CL488-28599	21017322	1:100

VIP	FITC	polyclonal	Proteintech	CL488-16233	21012083	1:100
CD45		30-F11	Invitrogen	14045182	2142894	1:200
E-cadherin		NCH-38	Thermoscientific	MA512547	ZD4272532	1:200
VIP		polyclonal	Thermoscientific	PA578224	ZD4273119	1:200
Substance P		polyclonal	Thermoscientific	PA5106934	ZD4263632	1:200
Goat anti mouse	Cy3		Jackson Immuno	115-165-003	130434	1:250
Goat anti rat	Alexa 647		Jackson Immuno	112-605-003	149594	1:250
Goat Anti Rabbit	Alexa 488	IgG H&L	Abcam	AB150077	GR3313703-1	1:250
Goat Anti mouse IgG HRP	HRP	polyclonal	Abcam	ab205719	1036603-21	log fold dilutios 1:10-1:10000
Goat Anti mouse IgE HRP	HRP	polyclonal	Thermofisher	PA1-84764	ZA4195726	log fold dilutios 1:10-1:10000
Goat serum			Abcam	AB7481	GR336429-10	2%
mABanti-Ly6g		1A8	Bioxcell	BE-0075-1	854523S1	150µg
IgG2a isotype control		2A3	Bioxcell	BE0089	849322J2	150µg
Antibody diluent			Bioxcell	IP0070	762123F1	

Table 3: Reagents for cell separation

Cell separation/preparation		
**Reagent**	**Vendor**	**Cat #**
Easy Sep Mouse B cell Kit	Stemcell technologies	19854A
Easy Sep Mouse CD138 positive selection kit	Stemcell technologies	18957
BD Cytofix/Cytoperm	BD Biosciences	554714
Brilliant Stain buffer	BD Biosciences	563794
Ultracomp beads	Invitrogen	01-2222-42
Spherotech Accucount beads		ACFP-70-5

Table 4: Reagents for histology

Histology		
**Reagent**	**Vendor**	**Cat #**
Giemsa	Abcam	AB150670
Periodic Acid Schiffs reagent kit	Sigma	395B-1Kt
Gram Stain	Sigma	HT90T-1KT
Eosin	Epredia	6766007
Hematoxylin	Epredia	6765001
Prolong Diamond Antifade	Thermofisher	P36961
Permount	Fisher	SP15100

Table 5: ELISA and luminex

ELISA/Luminex				
**Target (s)**	**ELISA/Luminex**	**Bead region (if applicable)**	**Vendor**	**Cat#**
TNFSF13	ELISA		Lifespan Biosciences	LS-F33759-1
IL-7	ELISA		R&D systems	DY407
IL-6	ELISA		R&D systems	DY406
CGRP	ELISA		Raybiotech	EIAM-CGRP-2
VIP	ELISA		Raybiotech	EIAM-VIP-2
Substance P	ELISA		Raybiotech	EIA-SP-2
NPY	ELISA		Raybiotech	EIAM-NPY-2
BAFF	Luminex	BR57	R&D systems	LXSAMSM-23
CCL5/Rantes	Luminex	BR38	R&D systems	LXSAMSM-23
CCL7/MCP-3	Luminex	BR39	R&D systems	LXSAMSM-23
CCL11/EOTAXIN	Luminex	BR74	R&D systems	LXSAMSM-23
CCL12/MCP	Luminex	BR42	R&D systems	LXSAMSM-23
CXCL13	Luminex	BR21	R&D systems	LXSAMSM-23
IFN-GAMMA	Luminex	BR33	R&D systems	LXSAMSM-23
IL1-ALPHA	Luminex	BR47	R&D systems	LXSAMSM-23
IL1-BETA	Luminex	BR19	R&D systems	LXSAMSM-23
IL-2	Luminex	BR22	R&D systems	LXSAMSM-23
IL-4	Luminex	BR25	R&D systems	LXSAMSM-23
IL-5	Luminex	BR26	R&D systems	LXSAMSM-23
IL-6	Luminex	BR27	R&D systems	LXSAMSM-23
IL-10	Luminex	BR28	R&D systems	LXSAMSM-23
IL-13	Luminex	BR29	R&D systems	LXSAMSM-23
IL-17A	Luminex	BR30	R&D systems	LXSAMSM-23
IL-33	Luminex	BR43	R&D systems	LXSAMSM-23
M-CSF	Luminex	BR45	R&D systems	LXSAMSM-23
RAGE	Luminex	BR78	R&D systems	LXSAMSM-23
S100A8	Luminex	BR20	R&D systems	LXSAMSM-23
P-SELECTIN	Luminex	BR46	R&D systems	LXSAMSM-23
SYNDECAN-1/CD138	Luminex	BR51	R&D systems	LXSAMSM-23
TNF-ALPHA	Luminex	BR14	R&D systems	LXSAMSM-23
IgA	Luminex	38	ThermoFisher	EPX070-20815-901
IgE	Luminex	42	ThermoFisher	EPX070-20815-901
IgG1	Luminex	12	ThermoFisher	EPX070-20815-901
IgG2a	Luminex	18	ThermoFisher	EPX070-20815-901

IgG2b	Luminex	21	ThermoFisher	EPX070-20815-901
IgG3	Luminex	25	ThermoFisher	EPX070-20815-901
IgM	Luminex	52	ThermoFisher	EPX070-20815-901
IgG	ELISA		ThermoFisher	88-50400-22
IgA	ELISA		ThermoFisher	88-50450-22
IgE	ELISA		ThermoFisher	EMIGHE

Table 6: Taqman probes and reagents

qPCR Taqman		
**Target/gene/reagent**	**Manufacturer**	**Assay ID/ Cat #**
VIP1R	Thermofisher	Mm00449214_m1
VIP2R	Thermofisher	Mm01238618_g1
Tac1R	Thermofisher	Mm00436892_m1
Tac2R	Thermofisher	Mm01175997_m1
NPY1R	Thermofisher	Mm00650798_g1
NPY2R	Thermofisher	Mm01956783_s1
NPY4R	Thermofisher	Mm00435894_s1
NPY5R	Thermofisher	Mm02620267_s1
NPY6R	Thermofisher	Mm00440546_s1
MRGPRA	Thermofisher	Mm01984314_s1
MRGPRB2	Thermofisher	Mm01956240_s1
MRGPRG	Thermofisher	Mm01701870_s1
RAMP1	Thermofisher	Mm00489796_m1
Tac1	Thermofisher	Mm01166996_m1
Calca	Thermofisher	Mm00801463_g1
VIP	Thermofisher	Mm00660234_m1
NPY	Thermofisher	Mm01410146_m1
TRPV1	Thermofisher	Mm01246300_m1
HPRT	Thermofisher	Mm03024075_m1
Taqman Fast Advance	Thermofisher	4444557
Tetro cDNA synthesis kit	Tetro biosystems	NC1352749
inTron Easy spin Total RNA extraction Kit	Boca Scientific	17221
Quantstudio 3	Thermofisher	A28567

Table 7: Chemicals list

Chemical, peptides & recombinant proteins		
**Reagent**	**Brand**	**Cat #**
Trtion x 100	Fisher	BP151-100
DMSO	Fisher	BP231-100
Collagenase IV	Sigma-Aldrich	C4-22-1g
Cytosine beta-D-arabinofuranoside	Sigma-Aldrich	C6645
B-27 Supplement (50X)	Thermo Fisher Scientific	10889038
GlutaMAX	GIBCO	35050061
Penicillin/Streptomycin	Sigma-Aldrich	P0781
HEPES	Sigma-Aldrich	H3662-100ML
Sodium Pyruvate	Fisher	11360070
Mercaptoethanol	GIBCO	21985-023
Alternaria alternata	CiteQ	09.01.26
Streptococcus Pneumoniaie	ATCC	49619
Brain Heart Media	Oxoid	CM1135
Agar	Sigma-Aldrich	70138
Tryptic Soy Agar	BD	236950
Tryptic soy broth	BD	211825
Todd Hewitt Broth	BD	249240
Yeast Extract	Fisher	50489152
Defibrinated Sheep Blood	Remel	R54012
Neomycin	Fisher	BP26695
IL4	Sigma-Aldrich	I1020-5UG
LPS	Sigma-Aldrich	L6529-1MG
Substance P	Tocris	1156
VIP	Tocris	1911
NPY	Tocris	1153
CGRP	Bachem	4025897.1
ACK Lysing Buffer	GIBCO	A10492-01
RPMI 1640	GIBCO	11835-030
Fetal Bovine Serum	Sigma-Aldrich	F8192
EDTA	Sigma-Aldrich	E6758-500G
BAFF	Tocris	8876-BF-010
Protease inhibitor cocktail	Tocris	5500
Resiniferatoxin	Adipogen	502053716
PBS	GIBCO	10010-023
HBSS	GIBCO	14175-095

Methacholine	MP Biomedicals	ICN19023110
Pancuronium bromide	MP Biomedicals	ICN15605350
Paraformaldehyde	Sigma-Aldrich	P6148
TMB substrate	Fisher Chemical	AAJ61325AP
H2SO4	Fisher Chemical	828016

Table 8: Software

Software	
**Software package/version**	**Manufacturer**
Prizm v10.0	Graphpad
BD Facs Diva8	BD Biosciences
Flowjo v10.0	Treestar
LASx	Leica

References

Tränkner, D., Hahne, N., Sugino, K., Hoon, M. A. & Zuker, C. Population of sensory neurons essential for asthmatic hyperreactivity of inflamed airways. *PNAS* 111, 11515–11520 (2014)
