# Supplementary material for: Sensory neurons regulate stimulus-dependent humoral immunity in mouse models of bacterial infection and asthma
Source: Nat Commun. 2024 Oct 16;15:8914. doi: 10.1038/s41467-024-53269-3 (PMC11484968; doi:10.1038/s41467-024-53269-3)
Supplement: Supplementary file 3 — Reporting Summary [file 41467_2024_53269_MOESM3_ESM.pdf]

nature portfolio

Corresponding author(s): Nicholas Jendzjowsky

Last updated by author(s): Sep 5, 2024

Reporting Summary

Nature Portfolio wishes to improve the reproducibility of the work that we publish. This form provides structure for consistency and transparency in reporting. For further information on Nature Portfolio policies, see our Editorial Policies and the Editorial Policy Checklist.

Statistics

For all statistical analyses, confirm that the following items are present in the figure legend, table legend, main text, or Methods section.

n/a	Confirmed	
☐	☒	The exact sample size (*n*) for each experimental group/condition, given as a discrete number and unit of measurement
☐	☒	A statement on whether measurements were taken from distinct samples or whether the same sample was measured repeatedly
☐	☒	The statistical test(s) used AND whether they are one- or two-sided<br>*Only common tests should be described solely by name; describe more complex techniques in the Methods section.*
☒	☐	A description of all covariates tested
☐	☒	A description of any assumptions or corrections, such as tests of normality and adjustment for multiple comparisons
☐	☒	A full description of the statistical parameters including central tendency (e.g. means) or other basic estimates (e.g. regression coefficient) AND variation (e.g. standard deviation) or associated estimates of uncertainty (e.g. confidence intervals)
☐	☒	For null hypothesis testing, the test statistic (e.g. *F*, *t*, *r*) with confidence intervals, effect sizes, degrees of freedom and *P* value noted<br>*Give P values as exact values whenever suitable.*
☒	☐	For Bayesian analysis, information on the choice of priors and Markov chain Monte Carlo settings
☒	☐	For hierarchical and complex designs, identification of the appropriate level for tests and full reporting of outcomes
☐	☒	Estimates of effect sizes (e.g. Cohen's *d*, Pearson's *r*), indicating how they were calculated

Our web collection on statistics for biologists contains articles on many of the points above.

Software and code

Policy information about availability of computer code

Data collection	Flow cytometry: BD FACSDiva Software V.8.0; Cytokine bead array: Luminex Xpotent Software V4.2; Plate reader: Gen5 version 3.11; Microscopy: LAS X Leica Application Suite X v 3.5.2.18963
Data analysis	GraphPad Prism (Version 10.0) FlowJo (Version 10.8.1) ImageJ (V1.8)

For manuscripts utilizing custom algorithms or software that are central to the research but not yet described in published literature, software must be made available to editors and reviewers. We strongly encourage code deposition in a community repository (e.g. GitHub). See the Nature Portfolio guidelines for submitting code & software for further information.

Data

Policy information about availability of data

All manuscripts must include a data availability statement. This statement should provide the following information, where applicable:

- Accession codes, unique identifiers, or web links for publicly available datasets
- A description of any restrictions on data availability
- For clinical datasets or third party data, please ensure that the statement adheres to our policy

The authors declare that the data supporting the findings of this study are available within the paper, the accompanying supplementary information files, and the source data (Source Data file).

Research involving human participants, their data, or biological material

Policy information about studies with human participants or human data. See also policy information about sex, gender (identity/presentation), and sexual orientation and race, ethnicity and racism.

Reporting on sex and gender	*Use the terms sex (biological attribute) and gender (shaped by social and cultural circumstances) carefully in order to avoid confusing both terms. Indicate if findings apply to only one sex or gender; describe whether sex and gender were considered in study design; whether sex and/or gender was determined based on self-reporting or assigned and methods used.*<br>*Provide in the source data disaggregated sex and gender data, where this information has been collected, and if consent has been obtained for sharing of individual-level data; provide overall numbers in this Reporting Summary. Please state if this information has not been collected.*<br>*Report sex- and gender-based analyses where performed, justify reasons for lack of sex- and gender-based analysis.*
Reporting on race, ethnicity, or other socially relevant groupings	*Please specify the socially constructed or socially relevant categorization variable(s) used in your manuscript and explain why they were used. Please note that such variables should not be used as proxies for other socially constructed/relevant variables (for example, race or ethnicity should not be used as a proxy for socioeconomic status).*<br>*Provide clear definitions of the relevant terms used, how they were provided (by the participants/respondents, the researchers, or third parties), and the method(s) used to classify people into the different categories (e.g. self-report, census or administrative data, social media data, etc.)*<br>*Please provide details about how you controlled for confounding variables in your analyses.*
Population characteristics	*Describe the covariate-relevant population characteristics of the human research participants (e.g. age, genotypic information, past and current diagnosis and treatment categories). If you filled out the behavioural & social sciences study design questions and have nothing to add here, write "See above."*
Recruitment	*Describe how participants were recruited. Outline any potential self-selection bias or other biases that may be present and how these are likely to impact results.*
Ethics oversight	*Identify the organization(s) that approved the study protocol.*

Note that full information on the approval of the study protocol must also be provided in the manuscript.

Field-specific reporting

Please select the one below that is the best fit for your research. If you are not sure, read the appropriate sections before making your selection.

☒ Life sciences ☐ Behavioural & social sciences ☐ Ecological, evolutionary & environmental sciences

For a reference copy of the document with all sections, see nature.com/documents/nr-reporting-summary-flat.pdf

Life sciences study design

All studies must disclose on these points even when the disclosure is negative.

Sample size	For in vivo experiments sample sizes were based on previous results (e.g.PMID: 34060477 and pilot experiments in our lab). In previous studies the response within each subject group was normally distributed with standard deviation 0.25. Therefore, our sample size calculations yielded n=5-8 with effect size of 0.5 and power of 0.8. The Type I error probability associated with this test of this null hypothesis was 0.05. All experiments used power analysis to determine the appropriate number of mice. At least three biological replicates were performed for all in vitro experiments unless otherwise indicated.
Data exclusions	No data were excluded.
Replication	All experimental findings were reproduced (in vivo: 2-4; in vitro;2-4 times).
Randomization	Mice were randomized to Vehicle or RTX treatment.
Blinding	Researchers were not blinded to the experimental groups because the endpoints (survival, bacterial burden, immunoglobulin levels, etc.) were objective measures of disease severity. However, the outcomes were quantitative and not subjective.

Reporting for specific materials, systems and methods

We require information from authors about some types of materials, experimental systems and methods used in many studies. Here, indicate whether each material, system or method listed is relevant to your study. If you are not sure if a list item applies to your research, read the appropriate section before selecting a response.

Materials & experimental systems

n/a	Involved in the study
	[x] Antibodies
[x]	Eukaryotic cell lines
[x]	Palaeontology and archaeology
	[x] Animals and other organisms
[x]	Clinical data
[x]	Dual use research of concern
[x]	Plants

Methods

n/a	Involved in the study
[x]	ChIP-seq
	[x] Flow cytometry
[x]	MRI-based neuroimaging

Antibodies

Antibodies used

AB Flurophore Clone Vendor Cat# Lot # Dilution
CD11c BV 421 N418 BIOLEGEND 117329 B366519 1:100
Ly-6G APC 1A8 BIOLEGEND 127614 B366717 1:100
Ly-6G BV421 RB6-8C5 BIOLEGEND 108433 B320343 1:100
CD11B PACIFIC BLUE M1/70 BIOLEGEND 101224 B375478 1:100
MHCII percp 5.5 M5/114.15.2 BIOLEGEND 107626 B354662 1:100
CD117 ckit PE 2B8 BIOLEGEND 105808 B343465 1:100
Siglec-F ALEXA 700 1RNM44N INVITROGEN 561702-82 2410787 1:100
F4 80 BV650 BM8 BIOLEGEND 123149 B326894 1:100
CD3 BUV805 17A2 BDBiosciences 569192 3047446 1:100
CD4 PEC594 GK1.5 BIOLEGEND 100455 B333712 1:100
CD8 BV711 SK1 BIOLEGEND 344734 B370263 1:100
T-BET APC 4B10 BIOLEGEND 644814 B353939 1:100
RORYT BV510 Q31-378 BDBiosciences 567177 3027175 1:100
B220 PercP 5.5 RA3-6B2 BIOLEGEND 103 236 B358603 1:100
B220 APC RA3-6B2 BIOLEGEND 103212 B379792 1:100
CD19 BV605 6D5 BIOLEGEND 115539 B386168 1:100
L-SELECTIN CD62L PE MEL-14 BIOLEGEND 104408 B374825 1:100
CD44 BV421 IM7 BIOLEGEND 103040 B393548 1:100
CD38 BUV395 90 INVITROGEN 363-0381-82 2774971 1:100
SYNDECAN-1 CD138 BV510 281-2 BIOLEGEND 142521 B374400 1:100
CD23 PE-Cy7 B3B4 BIOLEGEND 101614 B407653 1:100
IgD BV711 11-26C.2a BIOLEGEND 564275 3069422 1:100
IgG1 BV650 RMG1-1 BIOLEGEND 406629 B364333 1:100
IgM BUV805 IL41 BDBiosciences 749307 3219714 1:100
IgE ALEXA 488 RME-1 BIOLEGEND 406909 B368990 1:100
Ki67 BUV737 SolA15 INVITROGEN 367-5698-82 2653121 1:100
CD45 ALEXA 488 30-F11 BIOLEGEND 103122 B369434 1:100
Viability APC CY7 Invitrogen 65-0865-18 2836722 1:1000
CD16 32 TruStainFcx 93 BIOLEGEND 101320 B419152 1:100
VIP1R FITC AB_2341081 Alomone AVR-001-F-50UL AVR001FAN0150 1:100
TRPV1 AB_2313819 Alomone ACC-030 ACC030AG0440 1:200
MRGPRX PE-Cy7 K125H4 BIOLEGEND 359007 B393291 1:100
Tac1 FITC polyclonal Proteintech CL488-28599 21017322 1:100
VIP FITC polyclonal Proteintech CL488-16233 21012083 1:100
CD45 30-F11 Invitrogen 14045182 2142894 1:200
E-cadherin NCH-38 Thermoscientific MA512547 ZD4272532 1:200
VIP polyclonal Thermoscientific PA578224 ZD4273119 1:200
Substance P polyclonal Thermoscientific PA5106934 ZD4263632 1:200
Goat anti mouse Cy3 Jackson Immuno 115-165-003 130434 1:250
Goat anti rat Alexa 647 Jackson Immuno 112-605-003 149594 1:250
Goat Anti Rabbit Alexa 488 IgG H&L Abcam AB150077 GR3313703-1 1:250
Goat Anti mouse IgG HRP HRP polyclonal Abcam ab205719 1036603-21 log fold dilutios 1:10-1:10000
Goat Anti mouse IgE HRP HRP polyclonal Thermofisher PA1-84764 ZA4195726 log fold dilutios 1:10-1:10000
mABanti-Ly6g 1A8 Bioxcell BE-0075-1 854523S1 150μg
IgG2a isotype control 2A3 Bioxcell BE0089 849322J2

Validation

All antibodies were purchased from commercial sources. BD and BioLegend antibodies are tested by ELISA capture. Cell Signaling Technology Abs are tested for functionality, specificity, and sensitivity using siRNA and heterozygous knockout assays, mass spectrometry and in situ hybridization, IP, ChIP, and ChIP-seq, ELISA, peptide dot blots, peptide blocking, and protein arrays.

Animals and other research organisms

Policy information about studies involving animals; ARRIVE guidelines recommended for reporting animal research, and Sex and Gender in Research

Laboratory animals	C57BL/6J (#000664), Tac-/- (B6.Cg-Tac1tm1Bbm/J #004103), and µMT (B6.129S2-Ighmtm1Cgn/J #002288) mice were purchased from Jackson Laboratories. Vglut-TdTomato mice were provided by X. Sun (UCSD- crossed from Vglut2cre- and RosaTdTomato originally attained from JAX B6J.129S6(FVB)-Slc17a6tm2(cre)Lowl/MwarJ #028863 and B6.Cg-Gt(ROSA)26Sortm14(CAG-tdTomato)Hze/J #007914). TRPV1-DTR mice were obtained from Dr. Isaac Chiu, with MTA provided by M Hoon (NIH; PMID: 23536068). VIP1R-/- were kindly donated by Joseph Pisegna, Patrizia Germano, and James Waschek (UCLA, MGI: 177616 PMID: 21697765).
Wild animals	No wild animals were used in this study.
Reporting on sex	Male and female animals were used in this study.
Field-collected samples	No field collected samples were used in this study
Ethics oversight	All animal experiments were approved by The Lundquist Institute at Harbor UCLA Institutional Animal Care and Use Committee protocol # 32183.

Note that full information on the approval of the study protocol must also be provided in the manuscript.

Plants

Seed stocks	No plants were used.
Novel plant genotypes	None.
Authentication	None.

Flow Cytometry

Plots

Confirm that:

- [x] The axis labels state the marker and fluorochrome used (e.g. CD4-FITC).
- [x] The axis scales are clearly visible. Include numbers along axes only for bottom left plot of group (a 'group' is an analysis of identical markers).
- [x] All plots are contour plots with outliers or pseudocolor plots.
- [x] A numerical value for number of cells or percentage (with statistics) is provided.

Methodology

Sample preparation	The lungs were then dissected and flushed with PBS, coarse dissected, and incubated at 37°C for 45min in 1.75mg/ml collagenase IV (C4-22-1g Sigma) in PBS; then washed, macerated through a 21 gauge needle and filtered through a 70 µm mesh filter, then treated for flow cytometry. Spleens were macerated through a 70 µm mesh filter and then treated for flow cytometry. Bone marrow was flushed with RPMI media supplemented with 2mM EDTA and 10% FBS, filtered through a 70 µm mesh filter, then treated for flow cytometry.<br>Red blood cells were lysed with ACK lysing buffer (Gibco A10492-01), treated with Fc Block (Biolegend Trustain 101320), and resuspended in FACS buffer (HBSS-Gibco 10010-023 with 2% FBS Gibco. Incubations with antibody cocktails were conducted at 4°C for 60 min, and samples were subjected to two washes and resuspension in FACS buffer (HBSS +2% FBS Sigma F8192). For intracellular staining, cells were fixed/permeabilized with BD cytofix/cytoperm kit (554714), washed, and stained overnight at 4°C. Flow cytometry was conducted on a Symphony A5 flow cytometer (BD). Data were collected with BD DIVA software, and files were analyzed with FlowJo (Treestar, version 10.0.8r1). A live-cell stain (APC-Cy7, Invitrogen) was used to exclude dead cells. Positive staining and gates for each fluorescent marker were defined by comparing full stain sets with fluorescence minus one (FMO) control stain sets. All antibodies were 1:100. Antibodies used: Biolegend-CD11c (Clone N418), Ly6g (Clone 1A8), CD117 (Clone 2B8), Siglec F (Clone 1RNM44N Invitrogen), F4 80 (Clone BM8), CD3 (BUV805), CD3 (Clone17As), CD11c (Clone N48), CD4 (Clone GK1.5), CD8 (Clone SK1), T-bet (Clone 4B10), GATA3 (Clone 16E10A23), B220 (Clone RA3-6B2), CD19 (Clone 6D5), CD62L (Clone MEL-14), CD44 (Clone IM7), CD138 (Clone 281-2), IgG1 (Clone RMG1-1), IgE (Clone RME-1) CD45 (Clone 30-F11), MRGPRX (Clone K125H4); BDbiosciences- IgM (Clone IL41), IgD (Clone 11-26C.2a), RorγT (Clone Q31378; Invitrogen- CD38 (Clone 90), Ki67 (Clon SolA15); AlomoneLabs- VIP1R (Clone AB_2341081), Tac1 (polyclonal Proteintech), VIP (polyclonal Proteintech).

Instrument	BD FACSymphony™ A5 Cell Analyzer
Software	FACS Diva (BD Biosciences) and FlowJo software (Treestar)
Cell population abundance	Cells were enumerated with spherotech accucount counting beads. For B-cell cultures, cells were isolated with STEMCELL technologies EasySep B-cell negative selection kit. 90-95% purity was assessed as CD45+/CD3-/B220+.
Gating strategy	All in vivo cells were gated on leukocytes, singlets, live, CD45. Mast cells were CD117+MHCII+. Netrophils were Ly6g+, Eosinophils were SiglecF+CD11b+. T cells were divided into CD4+CD8- fractions and intracellularly stained for Tbet (Th1), or RORγT (γδ). B cell lineage were B220+CD19+. These were subdivided based on B Memory (CD44+CD38+), B Resident Memory (CD44+CD38+IgD-CD62L-), Isotype switched (IgM-IgD-). Plasma cells were CD138+, plasmablasts were CD138+B220lowCD19-.<br>For assessment of of either intracellular neuropeptides or neuropeptide receptors, cells were assessed from CD45+, live singlets and VIP or SP was stained intracellularly on CD3+, CD117+, Siglec F+, Ly6G+, or F480+ cells. VIPR1, MRGPRA1 was assessed on B220+CD19+ or CD138+ cells.<br>For in vitro cultures cells were divided into B220+CD19+ or CD138+. Then based on these gates assessed for IgG1 or IgE positivity. Ki67 was assessed from either CD138+IgG1+ or CD138+IgE+ gates. Exhaustion was assessed as CD11c+CD23- from CD138+ and B220+CD19+ gates.

☒ Tick this box to confirm that a figure exemplifying the gating strategy is provided in the Supplementary Information.
